# Chronic administration of pharmacological doses of angiotensin 1‐7 and iodoangiotensin 1‐7 has minimal effects on blood pressure, heart rate, and cognitive function of spontaneously hypertensive rats

**DOI:** 10.14814/phy2.14812

**Published:** 2021-04-27

**Authors:** Filipe F. Stoyell‐Conti, Alesa Chabbra, Joseph Puthentharayil, Katya Rigatto, Robert C. Speth

**Affiliations:** ^1^ College of Pharmacy Nova Southeastern University Fort Lauderdale FL USA; ^2^ Surgery Department University of Miami Miami FL USA; ^3^ Halmos College of Natural Science & Oceanography Nova Southeastern University Fort Lauderdale FL USA; ^4^ Institute for Neuro‐Immune Medicine Nova Southeastern University Fort Lauderdale FL USA; ^5^ Laboratório de Fisiologia Translacional Universidade Federal de Ciências da Saúde de Porto Alegre Porto Alegre RS Brazil; ^6^ Department of Pharmacology and Physiology College of Medicine Georgetown University Washington DC USA

**Keywords:** angiotensin 1‐7, angiotensin IV, blood pressure, cognition, heart rate, iodoAngiotensin 1‐7, memory, spontaneously hypertensive rat (SHR)

## Abstract

Cardiovascular diseases are the principal cause of death worldwide, with hypertension being the most common cardiovascular disease risk factor. High blood pressure (BP) is also associated with an increased risk of poor cognitive performance and dementia including Alzheimer's disease. Angiotensin 1–7 (Ang 1‐7), a product of the renin‐angiotensin system (RAS), exhibits central and peripheral actions to reduce BP. Recent data from our lab reveals that the addition of a non‐radioactive iodine molecule to the tyrosine in position 4 of Ang 1‐7 (iodoAng 1‐7) makes it ~1000‐fold more potent than Ang 1‐7 in competing for the ^125^I‐Ang 1‐7 binding site (Stoyell‐Conti et al., 2020). Moreover, the addition of the non‐radioactive iodine molecule increases (~4‐fold) iodoAng 1‐7’s ability to bind to the AT1 receptor (AT1R), the primary receptor for Ang II. Preliminary data indicates that iodoAng 1‐7 can also compete for the ^125^I‐Ang IV binding site with a low micromolar IC50. Thus, our aims were to compare the effects of chronic treatment of the Spontaneously Hypertensive Rat (SHR) with iodoAng 1‐7 (non‐radioactive iodine isotope) and Ang 1‐7 on arterial pressure, heart rate, and cognitive function. For this study, male SHRs were divided into three groups and treated with Saline, Ang 1‐7, or iodoAng 1‐7 administrated subcutaneously using a 28‐day osmotic mini pump. Systolic BP was measured non‐invasively by the tail‐cuff technique. Cognitive function was assessed by Y‐Maze test and novel object recognition (NOR) test. We have demonstrated in SHRs that subcutaneous administration of high doses of iodoAng 1‐7 prevented the increase in heart rate with age, while Ang 1‐7 showed a trend toward preventing the increase in heart rate, possibly by improving baroreflex control of the heart. Conversely, neither Ang 1‐7 nor iodoAng 1‐7 administered subcutaneously affected BP nor cognitive function.

## Graphical abstract

This study evaluated chronic high‐dose iodotyr(4)angiotensin 1‐7 and angiotensin 1‐7 administration on blood pressure, heart rate and cognitive function in SHR. Iodotyr(4)angiotensin 1‐7 and angiotensin 1‐7 prevented the increase in heart rate over a 27 day period but did not alter blood pressure or performance in a long‐term recognition test or a short‐term spatial memory task.

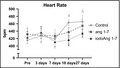

## 1 INTRODUCTION

Cardiovascular diseases are the principal cause of death worldwide, with hypertension the most common cardiovascular disease risk factor. Approximately 33% of Americans have hypertension and 36% have prehypertension. High blood pressure (BP) is also associated with an increased risk of poor cognitive performance and dementia including Alzheimer's disease (Iadecola et al. 2016). The role of the renin‐angiotensin system (RAS) in the regulation of BP, volume homeostasis, and the pathophysiology of hypertension has been studied for many decades (Manrique et al., 2009; Takimoto‐Ohnishi & Murakami, 2019). Increased RAS activity is also a major determinant for numerous pathologic conditions (Bavishi et al., 2016; Luft et al., 2012; Michel et al., 2016; Vadhan & Speth, 2020).

It is well documented that angiotensin II (Ang II) increases aldosterone and BP and contributes to the development of end‐organ damage through direct effects on cardiac, vascular, and renal tissues as well as impairment of cognitive function (Paul et al., 2006). On the other hand, Angiotensin 1–7 (Ang 1‐7), also a product of the RAS, exhibits central and peripheral actions to reduce BP and improve baroreflex sensitivity (Bennion et al., 2015; Gironacci et al., 2018; Iusuf et al., 2008), consistent with the concept that the ACE2/Ang 1‐7/Mas axis is a counter‐regulator of the ACE/Ang II/AT_1_R axis (Chappell et al., 2014; Paz Ocaranza et al., 2020), thus it may be a means to reduce high BP.

For more than a decade, Mas has been viewed as the receptor for Ang 1‐7. However, to date, the Mas receptor has not been pharmacologically characterized using radioligand binding in tissue membrane preparations. Our laboratory has demonstrated high affinity (low nanomolar KD) binding of ^125^I‐Ang 1‐7 in rat liver membranes, however, this binding is not pharmacologically specific in that the IC_50_ of Ang 1‐7 is in the micromolar range and all angiotensin peptides compete for ^125^I‐Ang 1‐7 binding equivalently (Stoyell‐Conti et al., 2020). Thus ^125^I‐Ang 1‐7 binding to liver, kidney, brain, and testes membrane preparations from both rats and mice is not a pharmacologically specific binding site for Ang 1‐7.

It is known that SHR has cognitive impairments (Meneses et al., 1996), even at young ages (Cao et al., 2012; Gattu et al., 1997; Grünblatt et al., 2015; Kantak et al., 2008; Tayebati et al., 2012). If these are a reflection of overactivity of the brain angiotensin system acting upon the AT1 receptor as suggested from radioligand binding assays (Gehlert et al., 1986; Gutkind et al., 1988) and mRNA determinations (Reja et al., 2006), then it is possible that Ang 1‐7 could reverse these cognitive impairments. It is known that hypertension is linked to damage to the BBB as recently reviewed (Setiadi et al., 2018). Given that SHR develops hypertension at the early age of 4–5 weeks (Dickhout & Lee, 1998; Harrap et al., 1990; Heijnen et al., 2014) and the blood‐brain‐barrier (BBB) is compromised in young SHR due to the high BP exerted upon the brain vasculature (Ueno et al., 2004), Ang 1‐7 may be able to enter the SHR brain to a greater extent than in normotensive rats.

It is important to note that in our receptor binding studies Ang 1‐7 which contains an iodine molecule on the tyrosine in position 4 of Ang 1‐7 may have different binding sites from Ang 1‐7. However, those experiments were performed in vitro and the physiological effects of iodoAng 1‐7 have not yet been studied in an animal model. Thus, considering that: 1) iodoAng 1‐7 is ~4‐fold more potent than Ang 1‐7 in competing for ^125^I‐SI‐Ang II binding to the AT1 receptor and 2) the addition of an iodine molecule to the tyrosine in position 4 of Ang 1‐7 makes it ~1000‐fold more potent than Ang 1‐7 in competing for the ^125^I‐Ang 1‐7 binding site (Stoyell‐Conti et al., 2020), we hypothesize that iodoAng 1‐7 can have antihypertensive effects and promote cognitive function to a greater extent than Ang 1‐7 in the spontaneously hypertensive rat (SHR) model of high BP and memory impairment. The SHR is a well‐characterized genetically‐determined animal model for hypertension (Doris, 2017; Folkow, 1982), permitting the study of the causes, mechanisms, and pathology of hypertension as well as the dysfunctions associated with it.

## 2 METHODS

### 2.1 Animal model

Eighteen male SHR (Charles River Laboratories), 11–12 weeks of age were housed in a temperature (22 ± 2°C) and humidity‐controlled (30%–40%) colony room maintained on a 12 h light:12 h dark cycle. Animals were allowed ad libitum access to chow and water. All animal experiments were carried out in accordance with the NIH guidelines for Use of Laboratory Animals and all procedures were performed under protocols approved by the Institutional Animal Care and Use Committee. The animal facility was accredited by the American Association for Accreditation of Laboratory Animal Care. Groups: Animals were randomly divided into three groups (n = 6): Saline (S), Ang 1‐7 (A), and iodoAng 1‐7 (IA).

### 2.2 Competition binding assay

Frozen rat brain tissues were thawed, mechanically homogenized in hypotonic buffer (20 mM NaPO4, pH 7.2), and centrifuged at 4oC (20,000× *g* for 20 min) to isolate membranes in the pellet. The membrane pellet was resuspended in the incubation buffer by rehomogenization at a concentration of 25–100 mg initial wet weight/ml of incubation buffer. The membrane homogenates were incubated with ^125^I‐Ang IV 1 nM with or without 5 varying concentrations of Ang IV or iodoAng 1‐7 for 60 min at room temperature (RT). The buffer used for the competition binding assay was 50 mM NaPO_4_, 150 mM NaCl, 5 mM EDTA, 0.1 mM bacitracin, pH 7.2. Membrane‐bound radioligand was separated from unbound radioligand by filtration over GF/B filters. Filter bound radioligand was analyzed with Graphpad PRISM software using a one‐site competition model: B = Bo *IC_50_/(IC_50_+ I), to derive the IC_50_ value where I is the competing ligand concentration, B = specifically bound radioligand and Bo is the amount of specific binding in the absence of competing ligand.

### 2.3 Subcutaneous implantation of the osmotic mini pump

Beginning at 11–12 weeks of age, Ang 1‐7, iodoAng 1‐7 or saline were administrated subcutaneously using a 28‐day osmotic mini pump (400 ng/kg/min) a dose used by previous investigators (Benter et al., 1995; Benter et al., 1995). The animals were anesthetized, shaved and their skin was washed over the implantation site. A mid‐scapular incision was made. A hemostat was inserted into the incision and the subcutaneous tissue was spread to create a pocket for the pump by opening and closing the jaws of the hemostat. The pocket was large enough to allow some free movement of the pump (e.g., 1 cm longer than the pump). The filled pump was inserted into the pocket with the delivery portal first. This minimizes interaction between the compound delivered and the healing of the incision. The incision was closed with sutures.

### 2.4 Non‐invasive blood pressure and heart rate measurement

The heart rate and BP were assessed in conscious rats by the tail‐cuff method using the BP‐2000 tail‐cuff system (Visitech, Raleigh, North Carolina) before and after the osmotic mini‐pump implantation. Prior to the beginning of the protocol, rats were adapted to the non‐invasive tail‐cuff plethysmography multi‐channel system three times a week for 2 weeks at the same time of day to measure systolic BP. This adaptation assures familiarity of the rats with the system, reduces stress levels, and promotes consistency in sequential readings (Gordish et al., 2017). Once the rats were adapted to the procedure, the experimental protocols were initiated using the exact same methods. A single experimenter was designated to conduct all measurements. BP was measured before the osmotic minipump implantation and every other day starting on the second day after the osmotic minipump implantation (Figure 2). To test this hypothesis, SHR animals were implanted with osmotic minipump and received saline, Ang 1‐7 or iodoAng 1‐7 for 28 days. BP and heart rate were measured pre and at 3, 7, 18, and 27 days after drug administration.

### 2.5 Y‐maze spontaneous alternation test

The Y‐maze test was used to measure spatial working memory (Sierksma et al., 2014). The apparatus consists of three identical arms (45 × 12 × 35 cm) diverging at a 120° angle with an equilateral triangular central area. Each animal was placed in the center of the Y‐maze and was free to explore the arena for 8 min. Rats tend to explore the least recently visited arm, and thus tend to alternate visits between the three arms. For efficient alternation, rats need to use working memory by maintaining an ongoing record of most recently visited arms and continuously updating such records (Wietrzych et al. 2005). An arm entry was scored when the rat placed its four paws within that arm. The following dependent variables were registered: total number of arm entries, number of triads (sequence of three consecutive visits to different arms), and percentage of alternation. An alternation was defined as an entry into three different arms on consecutive choices. The percentage of alternation was calculated as the ratio of actual to the maximum number of alternations. The maximum number of possible alternations was defined as the total number of arm entries minus 2. A low percentage of alternation is indicative of an impaired spatial working memory because the rat does not remember which arm it has just visited, and thus shows decreased spontaneous alternation. The Y‐maze test was performed at the end of the protocol.

### 2.6 Novel object recognition (NOR) test

The novel object recognition test was administered to assess non‐spatial, long‐term memory (Antunes & Biala, 2012; Martínez et al., 2014). Rats were placed in an open field 30 × 45 cm dimension in which two dissimilar objects were placed medially, 10 cm from the long ends of the open field. They were allowed to explore the environment and the objects placed in the open field for 5 minutes on day 1. One day later the rats were returned to the open field in which one of the objects had been replaced with a novel object. The rats were given 5 minutes to explore the environment again during which time their behavior was recorded with a digital camera. The time the rats spent exploring the novel object and the non‐novel object was assessed by 3 observers blinded to which object was which. The average of the scores of the observers was used to determine the time spent with each object, from which the percent of time spent exploring the novel object of the total time spent exploring the objects was determined.

**FIGURE 1:**
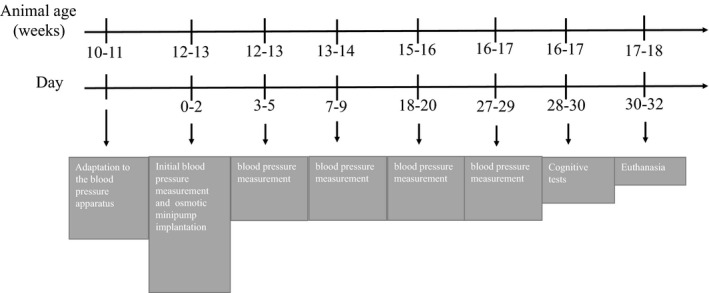
Timeline for the experimental protocol. Rats surgeries and BP measurements were staggered over a 3‐day period for groups of 6 rats (2 per group) such that measurements could be made during the first 6 hours of the light cycle. So, 6 rats had osmotic minipumps implanted on day 0, 6 on day 1 and 6 on day 2. The Y‐maze test was run on days 28–30. The NOR test was run on days 29–31

The timeline for these procedures is shown in Figure 1.

### 2.7 Statistical analysis

Data were expressed as mean ± SEM. The Shapiro‐Wilks test was used to evaluate data homogeneity. A one‐way or two‐way analysis of variance followed by Tukey's multiple comparison test was used to compare groups.

## 3 RESULTS

**FIGURE 2:**
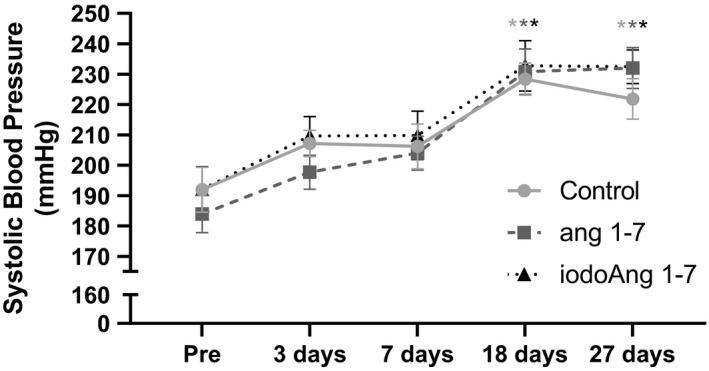
Systolic blood pressure in all studied groups at pre, 3, 7, 18, and 27 days of the protocol. Each * indicates *p* < 0.05 compared to day zero for all 3 groups.

**FIGURE 3:**
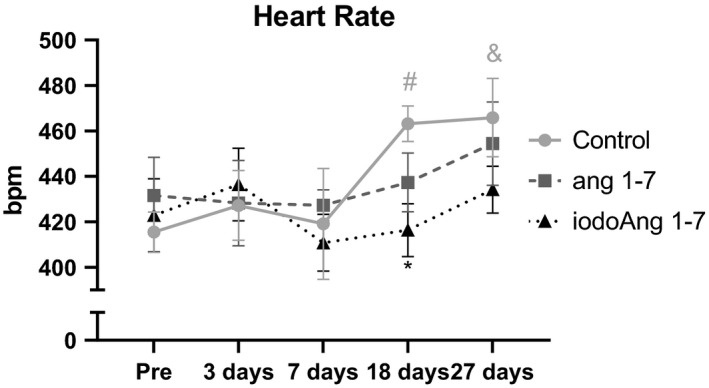
Heart rate in all studied groups at pre, 3, 7, 18, and 27 days of the protocol. #*p* < 0.05 vs. pre in the control group; *p < 0.05 vs. Control at 18 days; &*p* < 0.05 vs. day 3 in the control group

**FIGURE 4:**
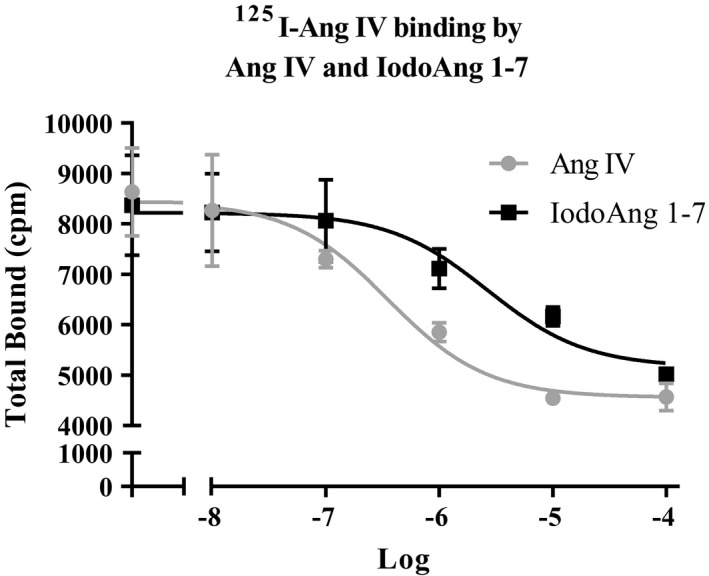
Competition binding assay for brain AT_4_ receptor binding of ^125^I‐Ang IV by Ang IV and iodoAng 1‐7 in the brain

**FIGURE 5:**
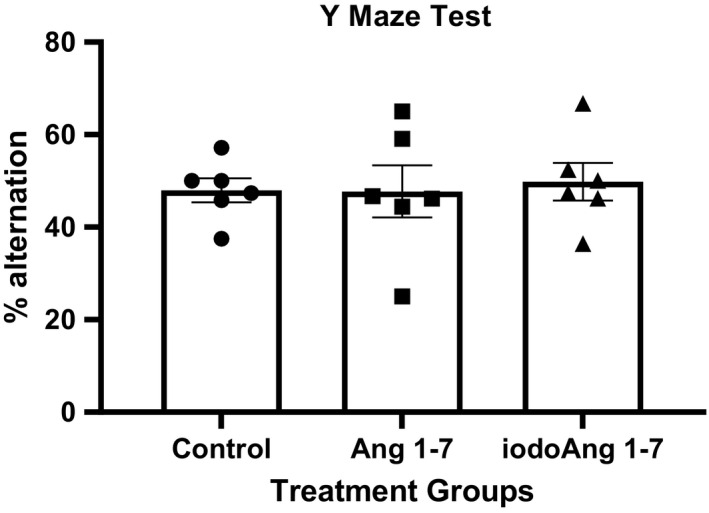
Y‐maze test results in the studied groups

**FIGURE 6:**
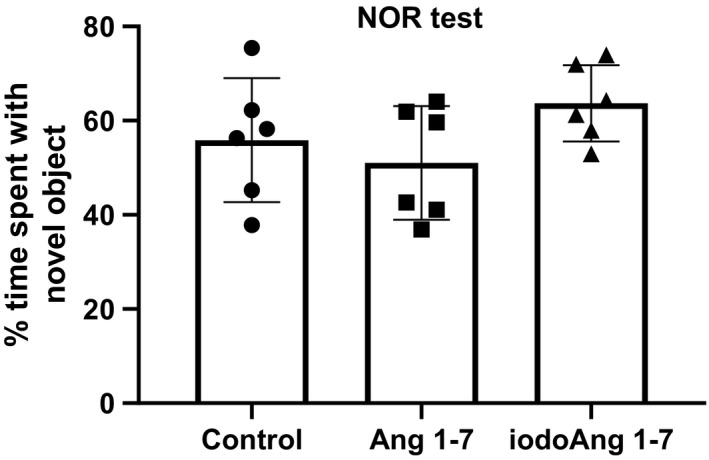
Novel object recognition test results in the studied groups

There was an increase in BP in all three groups over the course of the protocol with no difference among the studied groups (Figure 2). HR increased in the saline group during the protocol (3 vs. 27 days), however, both Ang 1‐7 and iodoAng 1‐7 prevented this increase. The iodoAng 1‐7 group presented lower HR than the saline group toward the end of the HR monitoring protocol (Figure 3). We also demonstrated that iodoAng 1‐7 competes for ^125^I‐Ang IV binding with a low micromolar IC_50_ (1.4 µM), suggesting that it could have actions at the AT_4_ receptor (Figure 4). As activation of the AT_4_ receptor has been associated with improved short‐term memory (Wright & Harding, 2004; Wright and Harding, 2008), we assessed the ability of Ang 1‐7 and iodoAng 1‐7 to improve spatial and short‐term memory. As shown in Figures 5 and 6, neither Ang 1‐7 nor iodoAng 1‐7 treatment affected cognitive performance as measured in the Y‐Maze (*F*_2,15_ = 0.59, *p* = 0.57) and NOR tests (*F*_2,15_ = 1.9, *p* = 0.18).

There were no significant changes in body weight or body weight gain of the different groups at the beginning or completion of the experiment (Table 1). The weights of the heart and kidney as well as the heart weight and kidney weight to body weight ratio did not differ between groups. The tissue weights of the brain, lungs, liver, intestines, colon, pancreas, spleen, adrenals epididymis, seminal vesicles, and adipose tissue also did not differ between groups (data not shown). However, there was a significant (*p* < 0.05) 17% increase in testis weight in the Ang 1‐7 treated rats compared to control rats (Table 1).

**TABLE 1:** Body and tissue weights (grams) of Control, Ang 1‐7‐treated and iodoAng 1‐7‐treated spontaneously hypertensive rats

Group	Pre‐treatment Body Weight	Post‐treatment Body Weight	Weight gain	Heart Weight	Kidney Weight	Testis weight
control	250 ± 18	298 ± 23	48	1.21 ± 0.11	1.90 ± 0.16	2.70 ± 0.17
Ang 1‐7	263 ± 33	313 ± 38	50	1.19 ± 0.11	2.00 ± 0.25	3.16 ± 0.09*
iodoAng 1‐7	258 ± 12	307 ± 12	49	1.22 ± 0.07	1.94 ± 0.20	2.87 ± 0.11

* *p* < 0.05 greater than control by Dunnett's multiple comparison test.

## 4 DISCUSSION

This is the first study to measure the effect of chronic iodoAng 1‐7 treatment on cardiovascular and cognitive function in the SHR and to compare it to an equivalent Ang 1‐7 treatment. While there were no significant effects of iodoAng 1‐7 and Ang 1‐7 treatment on BP, iodoAng 1‐7 reduced the increase in HR with age, while Ang 1‐7 showed a trend toward preventing the increase in HR, possibly improving peripherally the parasympathetic control to the heart which is inhibited by Ang II (Scroop & Lowe, 1969).

SHR is reported to perform poorly on the Y maze test compared to other rat strains although this may be attributed to their having an attention deficit hyperactivity disorder (ADHD) rather than impaired memory (Kishikawa et al., 2014; Yabuki et al., 2014; Yoon et al., 2013). In those studies WKY rats showed ~65–75% of spontaneous alternations while the SHR, or SHRSP (Yabuki et al., 2014) showed 55%, 55%, and 60%, respectively, which was slightly better than the 48, 47, and 50% spontaneous alternation than the control, Ang 1‐7 and iodoAng 1‐7 groups, respectively, seen in this study. The performance of SHRs in the NOR test is less clear. When tested 30 minutes after exposure to the novel objects, SHRs showed impaired performance on the NOR test (47% recognition of the novel object) compared to WKYs (60% recognition of the novel object) (Leffa et al., 2016). Stroke‐prone SHR also showed impaired novel object recognition in a 24 hour NOR test (32%) versus WKYs (67%) (Yabuki et al., 2014). However, in a 24‐hour NOR test, SHRs spent more time exploring the novel object compared to WKYs, which the authors attributed to an ADHD trait (dela Peña et al., 2015). In a 72‐hour interval test SHRs also showed better novel object recognition (67%) than the WKYs (61%) (Langen & Dost, 2011). The time spent examining the novel object by the rats in this study, 55%, 50.5%, and 65% for the control, Ang 1‐7, and iodoAng 1‐7 groups, respectively are within the range of the previous studies cited above. Whether the NOR test is assessing cognitive performance or ADHD behavior, our results show that neither experimental treatment altered this behavior.

Our results reinforce the statement that Ang 1‐7 is the most pleiotropic component of the RAS (Santos et al., 2000). It has been demonstrated that Ang 1‐7 can induce no change (Benter, Diz, et al., 1995; Botelho‐Santos et al., 2007; Campagnole‐Santos et al., 1992; Santos et al., 2004), an increase (Santos et al., 2000; Zhang et al., 2019) or a decrease (Iyer et al., 1998; Zhang et al., 2019) in BP depending on the dose, route of administration, animal strain, or the pathophysiological situation (Santos et al., 2000). For example, Ang 1‐7 can have different effects on modulating the BP and sympathetic activity depending on the site of infusion. Microinjection of Ang 1‐7 into the rostral ventrolateral medulla (RVLM), an important site in the brain that regulates sympathetic nervous system activity, increases mean arterial pressure and renal sympathetic nerve activity in renovascular hypertensive rats (Li et al., 2013).

There is a positive correlation between HR at 3‐wk of age and the level of elevated BP at 6‐wk, indicating the predictive value of elevated HR for the development of hypertension in the SHR (Dickhout & Lee, 1998). Therefore, the increase in the arterial BP in SHR is likely induced by a central sympathetic dominance that possibly opposes the important parasympathetic balance in these animals. Older SHR has normal sympathetic, but reduced vagal capacity to control HR in response to changes in mean arterial pressure; this deficit not being dependent on the absolute level of BP (Head & Adams, 1992). In any case, it is very important to consider that the parasympathetic nervous system presents a powerful vasodilatory mechanism for cerebral blood flow (Roloff et al., 2016). Because the SHR has reduced parasympathetic brainstem innervation (Roloff et al., 2018), this suggests that it may have a compromised vasodilatory capacity as well as vagal influence on heart rate. This could partially explain why the brainstem is severely hypoxic when BP is reduced to normal levels in SHRs (Marina et al., 2015) as the hypoxia stimulates sympathetic activity in an attempt to increase blood flow to the brainstem.

Systemic injection of ^125^I‐angiotensin II only reaches the Ang II receptors of the circumventricular organs that are not protected by the BBB (van Houten et al., 1980). Intravertebral artery administration of Ang II has been shown to act upon the area postrema, a circumventricular organ in the dorsal medulla to reduce parasympathetic activity (Joy & Lowe, 1970). It is likely that other circulating angiotensins also only reach BBB‐deficient brain regions (Roncevic, 2012). Thus, the same principle might apply to Ang 1‐7 and iodoAng 1‐7. But, if there are no receptors for Ang 1‐7 and iodoAng 1‐7 in circumventricular organs, then these peptides would not directly act upon the brain. Sustained hypertension is known to compromise the BBB (Setiadi et al., 2018), and, as the rats in this study were 11–12 weeks of age at the start of the experiment it is uncertain whether their BBB was still functioning normally, thereby affecting the ability of Ang 1‐7 or iodoAng 1‐7 to penetrate the BBB. Alternatively, blood‐borne angiotensin peptides have been shown to act indirectly on the brain by promoting nitric oxide (NO) formation by endothelial cells in the brain vasculature (Paton et al., 2008). This NO can vasodilate brain vasculature (Feterik et al., 2000) and enter the brain (Paton et al., 2008) to exert region‐specific effects on blood pressure regulation.

Mas receptor‐like immunoreactivity is reported to be abundant in the hippocampus, amygdala, anterodorsal thalamic nucleus, cortex, and hypoglossal nucleus in the rat brain, predominantly present in neurons, while Mas receptor mRNA expression is highest in the hippocampus (Young et al., 1988). However, a subsequent report has questioned the specificity of Mas receptor antibodies for Mas, so actual Mas protein expression in the hippocampus is still uncertain (Burghi et al., 2017). Central administration of Ang‐(1‐7) was found to exert various effects on the brain, such as enhancement of learning, memory, and cognitive performance (Wright & Harding, 2004). Additionally, a glycosylated Ang 1‐7 analog shown to penetrate the blood‐brain‐barrier was shown to reverse cognitive impairments in mice with experimental heart failure (Hay et al., 2019). While in the present study, we did not find any effects on cognitive response, this result is probably due to Ang 1‐7 and iodoAng 1‐7 not crossing the BBB.

The rat testis expresses a high level of mRNA for Mas (Metzger et al., 1995), the receptor for Ang 1‐7 (Santos et al., 2003). In the mouse, deletion of Mas causes a 33% reduction in testis weight (Leal et al., 2009). In human testis Mas mRNA expression is reduced in men with obstructive azoospermia, suggesting a functional role of Mas in male reproductive function (Reis et al., 2010). The increased testis weight seen in the Ang 1‐7 infused SHR in this study is consistent with a trophic function of Ang 1‐7 in the testis as a Mas agonist.

A limitation of our study is that an important component of this and any study of drug effects is the need to show a dose/response relationship for the experimental treatment. For this study, we were able to test only a single dose of Ang 1‐7 and iodoAng 1‐7. Considering that iodoAng 1‐7 is ~4‐fold more potent than Ang 1‐7 in competing for ^125^I‐SI‐Ang II binding to the AT1 receptor (Stoyell‐Conti et al., 2020) it is unlikely that there was any inhibition of AT_1_ receptor activity from iodoAng 1‐7 since blockade of AT_1_ receptors with angiotensin receptor blockers (ARBs) causes a reduction in systolic BP in the SHR (Sueta et al., 2014).

As new peptides are incorporated into the knowledge of the scientific community, the challenge to understand the role of RAS in the pathophysiology of diseases becomes greater. Thus, considering the complexity and number of new members of the RAS discovered in the past few years, the complex relationship between its peptides and receptors, where they interact, as well as their respective effects on the body, are far from being fully understood. Future investigations are required to clarify the role of RAS peptides in cardiovascular physiopathology.

## CONFLICT OF INTEREST

The authors report no conflict of interest.

## AUTHOR CONTRIBUTIONS

FFSC designed the experiments, conducted all of the experimental procedures, analyzed the data, co‐wrote the manuscript, and approved the final submission; AC assisted in the experimental procedures assisted in writing the manuscript and approved the final submission; JP assisted in the experimental procedures assisted in writing the manuscript and approved the final submission; KR analyzed the data, co‐wrote the manuscript and approved the final submission; RCS assisted in experimental design, assisted in the experimental procedures analyzed the data, co‐wrote the manuscript and approved the final submission.

## References

[phy214812-bib-0001] Antunes, M. , & Biala, G. (2012). The novel object recognition memory: neurobiology, test procedure, and its modifications. Cognitive Processing, 13(2), 93–110. 10.1007/s10339-011-0430-z 22160349 PMC3332351

[phy214812-bib-0002] Bavishi, C. , Bangalore, S. , & Messerli, F. H. (2016). Renin angiotensin aldosterone system inhibitors in hypertension: Is there evidence for benefit independent of blood pressure reduction? Progress in Cardiovascular Diseases, 59(3), 253–261. 10.1016/j.pcad.2016.10.002 27777044

[phy214812-bib-0003] Bennion, D. M. , Haltigan, E. , Regenhardt, R. W. , Steckelings, U. M. , & Sumners, C. (2015). Neuroprotective mechanisms of the ACE2‐angiotensin‐(1–7)‐Mas axis in stroke. Current Hypertension Reports, 17(2), 3. 10.1007/s11906-014-0512-2 25620630 PMC4378688

[phy214812-bib-0004] Benter, I. F. , Diz, D. I. , & Ferrario, C. M. (1995). Presser and reflex sensitivity is altered in spontaneously hypertensive rats treated with angiotensin‐(1–7). Hypertension, 26, 1138–1144. 7498984 10.1161/01.hyp.26.6.1138

[phy214812-bib-0005] Benter, I. F. , Ferrario, C. M. , Morris, M. , & Diz, D. I. (1995). Antihypertensive actions of angiotensin‐(1–7) in spontaneously hypertensive rats. American Journal of Physiology‐Heart and Circulatory Physiology, 269(1), H313–H319. 10.1152/ajpheart.1995.269.1.H313 7631863

[phy214812-bib-0006] Botelho‐Santos, G. A. , Sampaio, W. O. , Reudelhuber, T. L. , Bader, M. , Campagnole‐Santos, M. J. , & Souza dos Santos, R. A. (2007). Expression of an angiotensin‐(1–7)‐producing fusion protein in rats induced marked changes in regional vascular resistance. American Journal of Physiology. Heart and Circulatory Physiology, 292(5), H2485–H2490. 10.1152/ajpheart.01245.2006 17208987

[phy214812-bib-0007] Burghi, V. , Fernandez, N. C. , Gandola, Y. B. , Piazza, V. G. , Quiroga, D. T. , Guilhen Mario, E. , Felix Braga, J. , Bader, M. , Santos, R. A. S. , Dominici, F. P. , & Munoz, M. C. (2017). Validation of commercial Mas receptor antibodies for utilization in Western Blotting, immunofluorescence and immunohistochemistry studies. PLoS One, 12(8), e0183278. 28813513 10.1371/journal.pone.0183278 PMC5558983

[phy214812-bib-0008] Campagnole‐Santos, M. J. , Heringer, S. B. , Batista, E. N. , Khosla, M. C. , & Santos, R. A. (1992). Differential baroreceptor reflex modulation by centrally infused angiotensin peptides. American Journal of Physiology, 263(1 Pt 2), R89–R94. 10.1152/ajpregu.1992.263.1.R89 1636797

[phy214812-bib-0009] Cao, A. H. , Yu, L. , Wang, Y. W. , Wang, J. M. , Yang, L. J. , & Lei, G. F. (2012). Effects of methylphenidate on attentional set‐shifting in a genetic model of attention‐deficit/hyperactivity disorder. Behavioral and Brain Functions, 8(1), 10. 10.1186/1744-9081-8-10 22369105 PMC3312818

[phy214812-bib-0010] Chappell, M. C. , Marshall, A. C. , Alzayadneh, E. M. , Shaltout, H. A. , & Diz, D. I. (2014). Update on the Angiotensin converting enzyme 2‐Angiotensin (1–7)‐MAS receptor axis: fetal programing, sex differences, and intracellular pathways. Front Endocrinol (Lausanne), 4, 201. 10.3389/fendo.2013.00201 24409169 PMC3886117

[phy214812-bib-0011] dela Peña, I. , Gonzales, E. L. , de la Peña, J. B. , Kim, B.‐N. , Han, D. H. , Shin, C. Y. , & Cheong, J. H. (2015). Individual differences in novelty‐seeking behavior in spontaneously hypertensive rats: Enhanced sensitivity to the reinforcing effect of methylphenidate in the high novelty‐preferring subpopulation. Journal of Neuroscience Methods, 252, 48–54. 10.1016/j.jneumeth.2014.08.019 25169048

[phy214812-bib-0012] Dickhout, J. G. , & Lee, R. M. (1998). Blood pressure and heart rate development in young spontaneously hypertensive rats. American Journal of Physiology, 274(3), H794–H800. 10.1152/ajpheart.1998.274.3.H794 9530190

[phy214812-bib-0013] Doris, P. A. (2017). Genetics of hypertension: an assessment of progress in the spontaneously hypertensive rat. Physiological Genomics, 49(11), 601–617. 10.1152/physiolgenomics.00065.2017 28916635 PMC5792135

[phy214812-bib-0014] Feterik, K. , Smith, L. , & Katusic, Z. S. (2000). Angiotensin‐(1–7) causes endothelium‐dependent relaxation in canine middle cerebral artery. Brain Research, 873(1), 75–82. 10.1016/S0006-8993(00)02482-3 10915812

[phy214812-bib-0015] Folkow, B. (1982). Physiological aspects of primary hypertension. Physiological Reviews, 62(2), 347–504. 10.1152/physrev.1982.62.2.347 6461865

[phy214812-bib-0016] Gattu, M. , Pauly, J. R. , Boss, K. L. , Summers, J. B. , & Buccafusco, J. J. (1997). Cognitive impairment in spontaneously hypertensive rats: role of central nicotinic receptors. I. Brain Research, 771(1), 89–103. 10.1016/S0006-8993(97)00793-2 9383012

[phy214812-bib-0017] Gehlert, D. R. , Speth, R. C. , & Wamsley, J. K. (1986). Quantitative autoradiography of angiotensin II receptors in the SHR brain. Peptides, 7(6), 1021–1027. 10.1016/0196-9781(86)90132-4 3562315

[phy214812-bib-0018] Gironacci, M. M. , Vicario, A. , Cerezo, G. , & Silva, M. G. (2018). The depressor axis of the renin‐angiotensin system and brain disorders: a translational approach. Clinical Science (Lond), 132(10), 1021–1038. 10.1042/CS20180189 29802208

[phy214812-bib-0019] Gordish, K. L. , Kassem, K. M. , Ortiz, P. A. , & Beierwaltes, W. H. (2017). Moderate (20%) fructose‐enriched diet stimulates salt‐sensitive hypertension with increased salt retention and decreased renal nitric oxide. Physiological Reports, 5(7), e13162. 10.14814/phy2.13162 28408634 PMC5392503

[phy214812-bib-0020] Grünblatt, E. , Bartl, J. , Iuhos, D. I. , Knezovic, A. , Trkulja, V. , Riederer, P. , Walitza, S. , & Salkovic‐Petrisic, M. (2015). Characterization of cognitive deficits in spontaneously hypertensive rats, accompanied by brain insulin receptor dysfunction. Journal of Molecular Psychiatry, 3(1), 6. 10.1186/s40303-015-0012-6 26110057 PMC4479234

[phy214812-bib-0021] Gutkind, J. S. , Kurihara, M. , Castren, E. , & Saavedra, J. M. (1988). Increased concentration of angiotensin II binding sites in selected brain areas of spontaneously hypertensive rats. Journal of Hypertension, 6(1), 79–84. 10.1097/00004872-198801000-00012 3351297

[phy214812-bib-0022] Harrap, S. B. , Van der Merwe, W. M. , Griffin, S. A. , Macpherson, F. , & Lever, A. F. (1990). Brief angiotensin converting enzyme inhibitor treatment in young spontaneously hypertensive rats reduces blood pressure long‐term [see comments]. Hypertension, 16, 603–614. 10.1161/01.HYP.16.6.603 2246027

[phy214812-bib-0023] Hay, M. , Polt, R. , Heien, M. L. , Vanderah, T. W. , Largent‐Milnes, T. M. , Rodgers, K. , Falk, T. , Bartlett, M. J. , Doyle, K. P. , & Konhilas, J. P. (2019). A novel angiotensin‐(1–7) glycosylated mas receptor agonist for treating vascular cognitive impairment and inflammation‐related memory dysfunction. Journal of Pharmacology and Experimental Therapeutics, 369(1), 9–25. 10.1124/jpet.118.254854 PMC6413771 30709867

[phy214812-bib-0024] Head, G. A. , & Adams, M. A. (1992). Characterization of the baroreceptor heart rate reflex during development in spontaneously hypertensive rats. Clinical and Experimental Pharmacology and Physiology, 19(8), 587–597. 10.1111/j.1440-1681.1992.tb00509.x 1526065

[phy214812-bib-0025] Heijnen, B. F. J. , Van Essen, H. , Schalkwijk, C. G. , Janssen, B. J. A. , & Struijker‐Boudier, H. A. J. (2014). Renal inflammatory markers during the onset of hypertension in spontaneously hypertensive rats. Hypertension Research, 37(2), 100–109. 10.1038/hr.2013.99 23985702

[phy214812-bib-0067] Iadecola, C. , Yaffe, K. , Biller, J. , Bratzke, L. C. , Faraci, F. M. , Gorelick, P. B. , Gulati, M. , Kamel, H. , Knopman, D. S. , Launer, L. J. , Saczynski, J. S. , Seshadri, S. , & Zeki Al Hazzouri, A. (2016). Impact of hypertension on cognitive function: A scientific statement from the American Heart Association. Hypertension, 68(6), e67–e94. 27977393 10.1161/HYP.0000000000000053 PMC5361411

[phy214812-bib-0026] Iusuf, D. , Henning, R. H. , van Gilst, W. H. , & Roks, A. J. (2008). Angiotensin‐(1–7): Pharmacological properties and pharmacotherapeutic perspectives. European Journal of Pharmacology, 585(2–3), 303–312. 10.1016/j.ejphar.2008.02.090 18417117

[phy214812-bib-0027] Iyer, S. N. , Ferrario, C. M. , & Chappell, M. C. (1998). Angiotensin‐(1–7) contributes to the antihypertensive effects of blockade of the renin‐angiotensin system. Hypertension, 31(1 Pt 2), 356–361. 10.1161/01.HYP.31.1.356 9453328

[phy214812-bib-0028] Joy, M. D. , & Lowe, R. D. (1970). Evidence that the area postrema mediates the central cardiovascular response to angiotensin II. Nature, 228, 1303–1304. 10.1038/2281303a0 4321187

[phy214812-bib-0029] Kantak, K. M. , Singh, T. , Kerstetter, K. A. , Dembro, K. A. , Mutebi, M. M. , Harvey, R. C. , Deschepper, C. F. , & Dwoskin, L. P. (2008). Advancing the spontaneous hypertensive rat model of attention deficit/hyperactivity disorder. Behavioral Neuroscience, 122(2), 340–357. 10.1037/0735-7044.122.2.340 18410173

[phy214812-bib-0030] Kishikawa, Y. , Kawahara, Y. , Yamada, M. , Kaneko, F. , Kawahara, H. , & Nishi, A. (2014). The spontaneously hypertensive rat/Izm (SHR/Izm) shows attention deficit/hyperactivity disorder‐like behaviors but without impulsive behavior: therapeutic implications of low‐dose methylphenidate. Behavioral Brain Research, 274, 235–242. 10.1016/j.bbr.2014.08.026 25151620

[phy214812-bib-0031] Langen, B. , & Dost, R. (2011). Comparison of SHR, WKY and Wistar rats in different behavioural animal models: effect of dopamine D1 and alpha2 agonists. ADHD Attention Deficit and Hyperactivity Disorders, 3(1), 1–12. 10.1007/s12402-010-0034-y 21432613

[phy214812-bib-0032] Leal, M. C. , Pinheiro, S. V. , Ferreira, A. J. , Santos, R. A. , Bordoni, L. S. , Alenina, N. , Bader, M. , & França, L. R. (2009). The role of angiotensin‐(1–7) receptor Mas in spermatogenesis in mice and rats. Journal of Anatomy, 214(5), 736–743. 10.1111/j.1469-7580.2009.01058.x 19438767 PMC2707096

[phy214812-bib-0033] Leffa, D. T. , de Souza, A. , Scarabelot, V. L. , Medeiros, L. F. , de Oliveira, C. , Grevet, E. H. , Caumo, W. , de Souza, D. O. , Rohde, L. A. P. , & Torres, I. L. S. (2016). Transcranial direct current stimulation improves short‐term memory in an animal model of attention‐deficit/hyperactivity disorder. European Neuropsychopharmacology, 26(2), 368–377. 10.1016/j.euroneuro.2015.11.012 26792443

[phy214812-bib-0034] Li, P. , Sun, H. J. , Cui, B. P. , Zhou, Y. B. , & Han, Y. (2013). Angiotensin‐(1–7) in the rostral ventrolateral medulla modulates enhanced cardiac sympathetic afferent reflex and sympathetic activation in renovascular hypertensive rats. Hypertension, 61(4), 820–827. 10.1161/HYPERTENSIONAHA.111.00191 23424239

[phy214812-bib-0035] Luft, F. C. , Dechend, R. , & Müller, D. N. (2012). Immune mechanisms in angiotensin II‐induced target‐organ damage. Annals of Medicine, 44(Suppl 1), S49–54. 10.3109/07853890.2011.653396 22713149

[phy214812-bib-0036] Manrique, C. , Lastra, G. , Gardner, M. , & Sowers, J. R. (2009). The renin angiotensin aldosterone system in hypertension: roles of insulin resistance and oxidative stress. Medical Clinics of North America, 93(3), 569–582. 10.1016/j.mcna.2009.02.014 PMC2828938 19427492

[phy214812-bib-0037] Marina, N. , Ang, R. , Machhada, A. , Kasymov, V. , Karagiannis, A. , Hosford, P. S. , Mosienko, V. , Teschemacher, A. G. , Vihko, P. , Paton, J. F. , Kasparov, S. , & Gourine, A. V. (2015). Brainstem hypoxia contributes to the development of hypertension in the spontaneously hypertensive rat. Hypertension, 65(4), 775–783. 10.1161/HYPERTENSIONAHA.114.04683 25712724 PMC4354460

[phy214812-bib-0038] Martínez, M. C. , Villar, M. E. , Ballarini, F. , & Viola, H. (2014). Retroactive interference of object‐in‐context long‐term memory: role of dorsal hippocampus and medial prefrontal cortex. Hippocampus, 24(12), 1482–1492. 10.1002/hipo.22328 25044872

[phy214812-bib-0039] Meneses, A. , Castillo, C. , Ibarra, M. , & Hong, E. (1996). Effects of aging and hypertension on learning, memory, and activity in rats. Physiology and Behavior, 60(2), 341–345. 10.1016/S0031-9384(96)80002-3 8840889

[phy214812-bib-0040] Metzger, R. , Bader, M. , Ludwig, T. , Berberich, C. , Bunnemann, B. , & Ganten, D. (1995). Expression of the mouse and rat mas proto‐oncogene in the brain and peripheral tissues. FEBS Letters, 357(1), 27–32. 8001672 10.1016/0014-5793(94)01292-9

[phy214812-bib-0041] Michel, M. C. , Brunner, H. R. , Foster, C. , & Huo, Y. (2016). Angiotensin II type 1 receptor antagonists in animal models of vascular, cardiac, metabolic and renal disease. Pharmacology and Therapeutics, 164, 1–81. 10.1016/j.pharmthera.2016.03.019 27130806

[phy214812-bib-0042] Paton, J. F. , Wang, S. , Polson, J. W. , & Kasparov, S. (2008). Signalling across the blood brain barrier by angiotensin II: novel implications for neurogenic hypertension. Journal of Molecular Medicine, 86(6), 705–710. 18443753 10.1007/s00109-008-0324-4

[phy214812-bib-0043] Paul, M. , Poyan, M. A. , & Kreutz, R. (2006). Physiology of local renin‐angiotensin systems. Physiological Reviews, 86(3), 747–803. 10.1152/physrev.00036.2005 16816138

[phy214812-bib-0044] Paz Ocaranza, M. , Riquelme, J. A. , García, L. , Jalil, J. E. , Chiong, M. , Santos, R. A. S. , & Lavandero, S. (2020). Counter‐regulatory renin‐angiotensin system in cardiovascular disease. Nature Reviews Cardiology, 17(2), 116–129. 10.1038/s41569-019-0244-8 31427727 PMC7097090

[phy214812-bib-0045] Reis, A. B. , Araujo, F. C. , Pereira, V. M. , dos Reis, A. M. , Santos, R. A. , & Reis, F. M. (2010). Angiotensin (1–7) and its receptor Mas are expressed in the human testis: implications for male infertility. Journal of Molecular Histology, 41(1), 75–80. 10.1007/s10735-010-9264-8 20361351

[phy214812-bib-0046] Reja, V. , Goodchild, A. K. , Phillips, J. K. , & Pilowsky, P. M. (2006). Upregulation of angiotensin AT1 receptor and intracellular kinase gene expression in hypertensive rats. Clinical and Experimental Pharmacology and Physiology, 33(8), 690–695. 16895541 10.1111/j.1440-1681.2006.04420.x

[phy214812-bib-0070] Roloff, E. V. , Tomiak‐Baquero, A. M. , Kasparov, S. , & Paton, J. F. (2016). Parasympathetic innervation of vertebrobasilar arteries: Is this a potential clinical target? Journal of Physiology, 594(22), 6463–6485. 10.1113/JP272450 PMC5108906 27357059

[phy214812-bib-0047] Roloff, E. V. L. , Walas, D. , Moraes, D. J. A. , Kasparov, S. , & Paton, J. F. R. (2018). Differences in autonomic innervation to the vertebrobasilar arteries in spontaneously hypertensive and Wistar rats. Journal of Physiology, 596(16), 3505–3529. 10.1113/JP275973 PMC6092310 29797726

[phy214812-bib-0048] Roncevic, D. (2012). Does angiotensin II cross the blood‐brain barrier? Hypertension Research, 35(7), 775. 10.1038/hr.2012.55 22534521

[phy214812-bib-0049] Santos, R. A. , Campagnole‐Santos, M. J. , & Andrade, S. P. (2000). Angiotensin‐(1–7): an update. Regulatory Peptides, 91(1–3), 45–62. 10.1016/S0167-0115(00)00138-5 10967201

[phy214812-bib-0050] Santos, R. A. , Ferreira, A. J. , Nadu, A. P. , Braga, A. N. , de Almeida, A. P. , Campagnole‐Santos, M. J. , Baltatu, O. , Iliescu, R. , Reudelhuber, T. L. , & Bader, M. (2004). Expression of an angiotensin‐(1–7)‐producing fusion protein produces cardioprotective effects in rats. Physiological Genomics, 17(3), 292–299. 10.1152/physiolgenomics.00227.2003 15039487

[phy214812-bib-0051] Santos, R. A. S. , Silva, A. C. S. E. , Maric, C. , Silva, D. M. R. , Machado, R. P. , de Buhr, I. , Heringer‐Walther, S. , Pinheiro, S. V. B. , Lopes, M. T. , Bader, M. , Mendes, E. P. , Lemos, V. S. , Campagnole‐Santos, M. J. , Schultheiss, H. P. , Speth, R. , & Walther, T. (2003). Angiotensin‐(1–7) is an endogenous ligand for the G protein‐coupled receptor Mas. Proceedings of the National Academy of Sciences of the United States of America, 100(14), 8258–8263. 10.1073/pnas.1432869100 12829792 PMC166216

[phy214812-bib-0052] Scroop, G. C. , & Lowe, R. D. (1969). Efferent pathways of the cardiovascular response to vertebral artery infusions of angiotensin in the dog. Clinical Science, 37, 605–619. 4312146

[phy214812-bib-0053] Setiadi, A. , Korim, W. S. , Elsaafien, K. , & Yao, S. T. (2018). The role of the blood‐brain barrier in hypertension. Experimental Physiology, 103(3), 337–342. 10.1113/EP086434 28986948

[phy214812-bib-0054] Sierksma, A. S. , van den Hove, D. L. , Pfau, F. , Philippens, M. , Bruno, O. , Fedele, E. , Ricciarelli, R. , Steinbusch, H. W. , Vanmierlo, T. , & Prickaerts, J. (2014). Improvement of spatial memory function in APPswe/PS1dE9 mice after chronic inhibition of phosphodiesterase type 4D. Neuropharmacology, 77, 120–130. 10.1016/j.neuropharm.2013.09.015 24067928

[phy214812-bib-0055] Stoyell‐Conti, F. F. , Itty, S. , Abraham, C. , Rigatto, K. , West, C. A. , & Speth, R. C. (2020). 125I‐Angiotensin 1–7 binds to a different site than angiotensin 1–7 in tissue membrane preparations. Endocrine, 1–7. 10.1007/s12020-020-02572-2 33415576

[phy214812-bib-0068] Sueta, D. , Koibuchi, N. , Hasegawa, Y. , Toyama, K. , Uekawa, K. , Katayama, T. , Ma, M. , Nakagawa, T. , Waki, H. , Maeda, M. , Ogawa, H. , & Kim‐Mitsuyama, S. (2014). Blood pressure variability, impaired autonomic function and vascular senescence in aged spontaneously hypertensive rats are ameliorated by angiotensin blockade. Atherosclerosis, 236(1), 101–107. 25016364 10.1016/j.atherosclerosis.2014.06.016

[phy214812-bib-0056] Takimoto‐Ohnishi, E. , & Murakami, K. (2019). Renin‐angiotensin system research: from molecules to the whole body. The Journal of Physiological Sciences, 69(4), 581–587. 10.1007/s12576-019-00679-4 31028527 PMC10717639

[phy214812-bib-0057] Tayebati, S. K. , Tomassoni, D. , & Amenta, F. (2012). Spontaneously hypertensive rat as a model of vascular brain disorder: microanatomy, neurochemistry and behavior. Journal of the Neurological Sciences, 322(1–2), 241–249. 22726353 10.1016/j.jns.2012.05.047

[phy214812-bib-0058] Ueno, M. , Sakamoto, H. , Tomimoto, H. , Akiguchi, I. , Onodera, M. , Huang, C. L. , & Kanenishi, K. (2004). Blood‐brain barrier is impaired in the hippocampus of young adult spontaneously hypertensive rats. Acta Neuropathologica, 107(6), 532–538. 10.1007/s00401-004-0845-z 15042385

[phy214812-bib-0059] Vadhan, J. D. , & Speth, R. C. (2020). The role of the brain renin‐angiotensin system (RAS) in mild traumatic brain injury (TBI). Pharmacology and Therapeutics, 218, 107684. 10.1016/j.pharmthera.2020.107684 32956721

[phy214812-bib-0060] van Houten, M. , Schiffrin, E. L. , Mann, J. F. E. , Posner, B. I. , & Boucher, R. (1980). Radioautographic localization of specific binding sites for blood‐borne angiotensin II in the rat brain. Brain Research, 186, 480–485. 10.1016/0006-8993(80)90995-6 6244062

[phy214812-bib-0069] Wietrzych, M. , Meziane, H. , Sutter, A. , Ghyselinck, N. , Chapman, P. F. , Chambon, P. , & Krezel, W. (2005). Working memory deficits in retinoid X receptor gamma‐deficient mice. Learning & Memory, 12(3), 318–326. 15897255 10.1101/lm.89805 PMC1142461

[phy214812-bib-0061] Wright, J. W. , & Harding, J. W. (2004). The brain angiotensin system and extracellular matrix molecules in neural plasticity, learning, and memory. Progress in Neurobiology, 72(4), 263–293. 10.1016/j.pneurobio.2004.03.003 15142685

[phy214812-bib-0062] Wright, J. W. , & Harding, J. W. (2008). The angiotensin AT4 receptor subtype as a target for the treatment of memory dysfunction associated with Alzheimer's disease. Journal of the Renin‐Angiotensin‐Aldosterone System, 9(4), 226–237. 19126664 10.1177/1470320308099084

[phy214812-bib-0063] Yabuki, Y. , Shioda, N. , Maeda, T. , Hiraide, S. , Togashi, H. , & Fukunaga, K. (2014). Aberrant CaMKII activity in the medial prefrontal cortex is associated with cognitive dysfunction in ADHD model rats. Brain Research, 1557, 90–100. 10.1016/j.brainres.2014.02.025 24561222

[phy214812-bib-0064] Yoon, S. Y. , dela Peña, I. , Kim, S. M. , Woo, T. S. , Shin, C. Y. , Son, K. H. , Park, H. , Lee, Y. S. , Ryu, J. H. , Jin, M. , Kim, K.‐M. , & Cheong, J. H. (2013). Oroxylin A improves attention deficit hyperactivity disorder‐like behaviors in the spontaneously hypertensive rat and inhibits reuptake of dopamine in vitro. Archives of Pharmacal Research, 36(1), 134–140. 10.1007/s12272-013-0009-6 23371806

[phy214812-bib-0065] Young, D. , O'Neill, K. , Jessell, T. , & Wigler, M. (1988). Characterization of the rat mas oncogene and its high‐level expression in the hippocampus and cerebral cortex of rat brain. Proceedings of the National Academy of Sciences of the United States of America, 85(14), 5339–5342. 2455902 10.1073/pnas.85.14.5339 PMC281746

[phy214812-bib-0066] Zhang, F. , Xu, Y. , Pan, Y. , Sun, S. , Chen, A. , Li, P. , Bao, C. , Wang, J. , Tang, H. , & Han, Y. (2019). Effects of Angiotensin‐(1–7) and Angiotensin II on Acetylcholine‐Induced Vascular Relaxation in Spontaneously Hypertensive Rats. Oxidative Medicine and Cellular Longevity, 2019, 6512485. 10.1155/2019/6512485 31827689 PMC6886389

